# Acceptability of insecticide-treated clothing for malaria prevention among migrant rubber tappers in Myanmar: a cluster-randomized non-inferiority crossover trial

**DOI:** 10.1186/s12936-017-1737-8

**Published:** 2017-02-28

**Authors:** Alison F. Crawshaw, Thae Maung Maung, Muhammad Shafique, Nyan Sint, Sarala Nicholas, Michelle S. Li, Arantxa Roca-Feltrer, Jeffrey Hii

**Affiliations:** 1 Malaria Consortium Myanmar, 37/B Thiri Mingalar Street, Kamayut Township, Yangon, Myanmar; 2 Department of Medical Research, Ministry of Health and Sports, No. 5, Ziwaka Road, Dagon Township, Yangon, Myanmar; 3 Malaria Consortium Asia, Faculty of Tropical Medicine, Mahidol University, Santasiri Sommani Building, 8th Floor, 420/6 Rajavidhi Road, Bangkok, 10400 Thailand; 4 Vector Borne Diseases Control (National Malaria Control Programme), Mon State Public Health Department, Ministry of Health and Sports, Science School Street, Bo Gone Quarter, Mawlamyine, Mon State Myanmar; 5 Malaria Consortium, 56 Leonard Street, London, EC2A 4LT UK

**Keywords:** Acceptability, Insecticide-treated clothing, Rubber tappers, Personal protection, Outdoor transmission

## Abstract

**Background:**

Insecticide-treated clothing (ITC) has long been used for military and outdoor recreational purposes and there is substantial evidence to show that it can protect against arthropod biting. As a complementary vector control measure, ITC could be used to address outdoor transmission of malaria, particularly among mobile and migrant populations and night-time workers such as rubber tappers, who may be beyond the reach of core interventions. However, more information is required on acceptability and preferences of target groups towards ITC to understand whether it could be a viable strategy in Myanmar.

**Methods:**

A cluster-randomized, double-blind, non-inferiority crossover trial was performed to determine acceptability of ITC versus identical, untreated clothing (NTC) among migrant rubber tappers. The study took place between January and May 2015 with 234 participants in 16 clusters in Thanbyuzayat Township, Mon State, Myanmar. Participants were randomly assigned to the order of clothing distribution and followed up at 2, 4 and 6 week intervals. Acceptability was assessed through structured questionnaires, focus group discussions and in-depth interviews. A cluster-level non-inferiority analysis was conducted using STATA, while qualitative data were digitally recorded, transcribed and content-analysed to identify patterns and themes, and managed thematically in Excel 2010^®^.

**Results:**

Acceptability of both types of clothing was high. ITC was deduced to be non-inferior to NTC for seven out of eight indicators regarding perceptions (looks nice, is durable, is pleasant to wear for nighttime work, reduces mosquito bites, would recommend the clothing, would buy the clothing, like the clothing overall). A high proportion of respondents reported that the clothing reduced mosquito bites (ITC-98%; NTC-94%). Clothing was worn regularly (about 11 times in the previous two weeks). The most common reasons for not wearing the clothing every night were that it was being washed or dried, or the participant did not go to work.

**Conclusions:**

The high level of acceptability suggests that ITC could be an appropriate strategy for personal protection amongst migrant rubber tappers in outdoor transmission settings in Myanmar. However, more research is needed into the feasibility and protective efficacy of ITC before it can be considered for wider roll-out.

*Trial registration* Clinical trials ACTRN12615000432516

## Background

Myanmar has a long history of reporting the highest malaria morbidity and mortality rates in both the WHO South-East Asia Region and the Greater Mekong Sub-Region (GMS) [1]. The country accounts for over 70 per cent of cases in the GMS [2] and reported 152,195 confirmed cases in 2015 [3]. With the recent scale-up of malaria prevention and control measures, there has been a visible decline in malaria morbidity from 11.2 cases per 1000 population in 2005 to 4.1 per 1000 population in 2014, and a corresponding decline in mortality from 1707 to 92 deaths, respectively [2]. Malaria infection is now largely concentrated among hard-to-reach and other risk groups, including mobile and migrant populations, forest-goers and outdoor night-time workers (including rubber tappers), who are exposed to outdoor biting vectors [1, 2].

The current core interventions for malaria prevention and control in Myanmar comprise long-lasting insecticidal nets (LLIN), indoor residual spraying (IRS), early diagnosis and treatment and behaviour change activities [2]. Myanmar’s transmission reduction programme relies almost entirely on achieving coverage with LLIN applied indoors and continuing reliance on bed nets alone will have variable impact on transmission, according to the habits of mosquitoes and humans. Recent data on insecticide resistance from the National Malaria Control Programme (NMCP) show high sensitivity to the pyrethroid insecticides used on LLIN [2].

The risk of exposure to outdoor transmission of malaria is increased by taking part in outdoor night-time activities such as forest-going, mining and plantation-related activities, where people are not covered by core interventions (such as bed nets and IRS) and may also have more limited access to preventative measures. In Myanmar, rubber tapping is a key resource sector and attracts a large migrant workforce. Rubber tapping takes place throughout the night, coinciding with peak biting times of *Anopheles dirus* and *Anopheles minimus*, the primary vectors in Myanmar [4, 5], and providing the opportunity for host–vector interactions.

Alternative vector control measures are needed where people are beyond the reach of core interventions. Several complementary measures to LLIN have been put forward with outdoor transmission in mind, including spatial and topical repellents, toxic sugar baits and insecticide-treated hammock nets [6–10]. However, current tools such as topical repellents have had limited impact at the community level [11–13] and a recent systematic review and meta-analysis concluded that topical repellents are not protective against falciparum or vivax malaria [10, 14]. It is thought that these tools are vulnerable to failure due to the behavioural change required; adherence to topical repellents is difficult as it requires regular re-application every few hours [11–13, 15] and maintenance of an intensive distribution scheme [6]. A strategy such as insecticide-treated clothing (ITC) may be more successful, given that individuals are already accustomed to wearing long clothing for night-time outdoor work (Muhammad Shafique, personal communication, 2013); [16, 17].

To date, use of ITC has been mostly limited to military, wildlife, wetland and park workers and outdoor recreational markets [18–21]. There is substantial evidence that ITC and treated-materials can prevent arthropod biting (including mosquitoes, ticks, chiggers), and vector borne diseases such as leishmaniasis [22, 23]. Wearing permethrin-ITC has been shown to reduce *Aedes* biting rates by more than ninety per cent [22]. For malaria prevention, results have ranged from showing no demonstrated reduction in incidence in Thailand [24] to up to 64% reduction in Afghanistan [25] and 69% reduction or more in Kenya [26–28]. However, none of these studies have systematically investigated acceptability and preference of ITC, which is important for uptake, targeted distribution and sustainability of the strategy, and may be a factor in the wide variability in results.

A recent study in Thailand showed high acceptability for insecticide-treated school uniforms for dengue prevention [29], but so far, preference and acceptability of ITC has not been evaluated among high risk groups for malaria prevention. Given these differences (settings, population, disease), it will be important to assess users’ perceptions and acceptability of ITC among the mobile, migrant rubber tapper population in Myanmar.

The primary objective of the study was thus to investigate preference and acceptability of ITC for malaria prevention among migrant rubber tappers in a malaria endemic area.

## Methods

### Study area

**Fig. 1:**
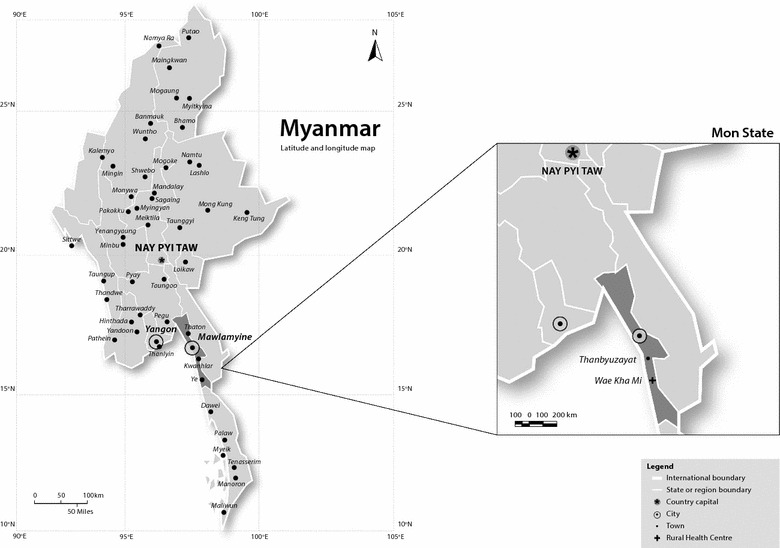
Map showing region of study area

The study was conducted in Wae Kha Mi Rural Health Centre (RHC), located in Thanbyuzayat Township, Mon State, southeastern Myanmar (Fig. 1). Wae Kha Mi RHC catchment area had a population of approximately 16,041 in 2015 with annual malaria parasite incidence (API) of 7.6 per thousand [30]. The landscape is characterized by forested foothills and plains which are dominated by rubber plantations, and which attract a large number of seasonal migrant workers for work. A scoping visit to Wae Kha Mi conducted in 2013 provided basic information on the study area, population and community practices and preferences for the study (Muhammad Shafique, pers. comm., 2013).

Mon State has a tropical climate and temperate weather, due to its location near the sea and in the low latitude zone. In the state capital of Mawlamyine, the average temperature in January is 25.6 °C and in April is 29.4 °C, with annual rainfall of 4826 mm. Rain is especially heavy in July and August.

### Vector ecology in Mon State

*Anopheles dirus* is a widespread species in Mon state and is especially found in forest and forested foot hill areas. In the nearby township of Mudon, approximately 40 km north of Thanbyuzayat, *An. minimus* and *Anopheles maculatus* were also found, but their numbers were low compared to *An. dirus* which was collected throughout the year [4]. *Anopheles dirus* appears to be responsible for perennial malaria transmission in the Mudon community especially in the post-monsoon months (i.e. September and October). Malaria incidence showed two distinct peaks corresponding to the months of June to July (peak monsoon season) and December to February (cool and dry season). Rubber tapping season is from November to May during the dry season, and ceases during the monsoon season, which is characterized by the outward migration of some workers. Shaded domestic wells provide excellent breeding habitats for *An. dirus* in Mudon area, contributing to high larval and pupal density during the rainy season, and low numbers during the cool-dry season [4]. This ecological adaptation in human settlements and shaded habitats may contribute to outdoor transmission among rubber tappers.

### Study design

A two-arm non-inferiority, cluster-randomized, double-blind crossover trial was conducted to determine whether acceptability of ITC was non-inferior to non-treated clothing (NTC). Participants and data collectors were blinded to the intervention. The study used mixed methods; quantitative components comprised a household survey and three structured questionnaires, and qualitative components comprised focus group discussions (FGD) and in-depth interviews (IDI). All study tools were developed in English, translated to Burmese and back translated for quality control. The study reported here was conducted from January to May 2015, during the dry season.

### Sampling framework

**Fig. 2:**
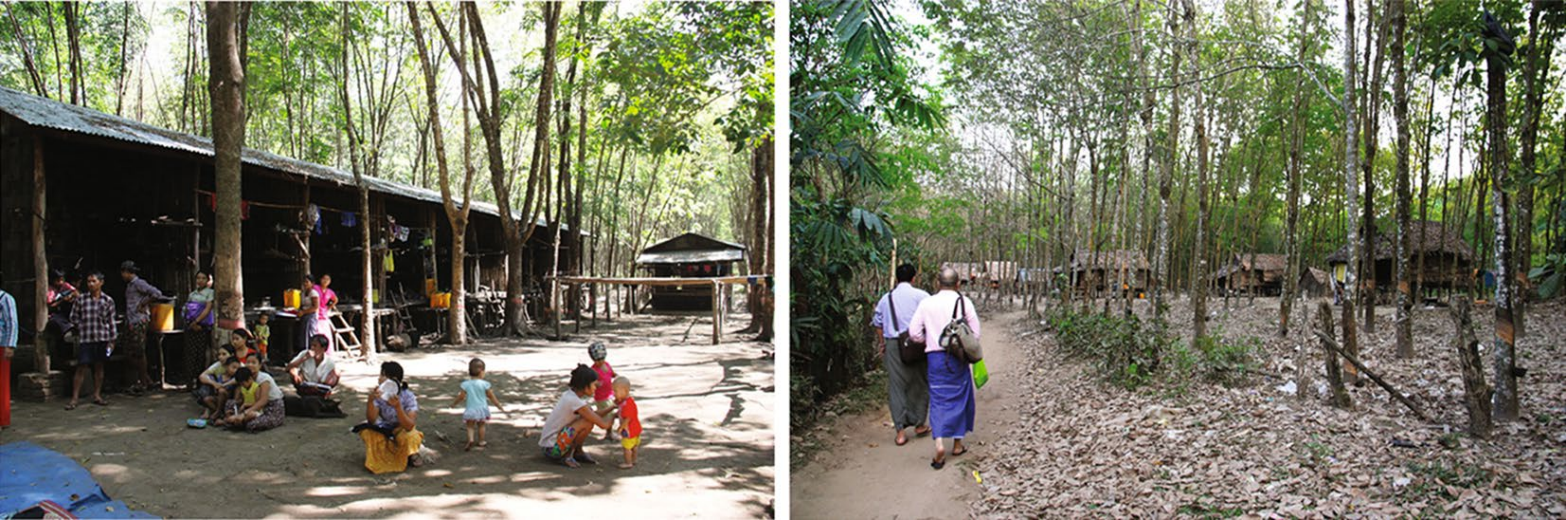
Different types of households in the study area; stilted, longhouse style lodging, and free-standing thatched houses on stilts

A cluster was defined as a discernible group of houses within a large rubber plantation (≥12 households), or a cluster of households in contiguous rubber plantations. Clusters were selected from household listings of all rubber plantations in the entire study area. After an initial baseline survey with all households identified in the study area, social mapping was used to define clusters according to the geographical positions of the plantations, numbers of households and numbers of rubber tappers (Fig. 2). Cluster size varied from 6 to 20 households and the number of eligible participants in each cluster varied from 10 to 37. Clusters were randomly allocated to the two study arms. Because of the variability in cluster size, the female participants were only randomly selected for enrolment by probability proportional to size (PPS) sampling. For the male participants’ enrolment, all the eligible participants were enrolled except for in three clusters whose size allowed random selection.

### Study inclusion

The study population comprised migrant rubber tappers who met the following inclusion criteria: reside or lodge (overnight) in the rubber plantation (situated outside the village limits) and answer “yes” to the question, “Are you a migrant?”; intend to stay in the study area for greater than or equal to four months; male or female aged 18 years or older; any nationality or ethnicity; give informed consent; working as rubber tappers overnight in the plantation. The exclusion criteria were: plantation workers not doing rubber tapping activities; reside outside of the rubber plantation (i.e. only coming to the plantation for rubber tapping); registered in the village population register; answer “no” to the question, “Are you a migrant?”; known skin allergy or past history of a reaction to LLIN (self-reported); pregnant or breastfeeding women.

### Sample size

#### Quantitative

Sample size was powered to allow pairwise comparison between ITC and NTC, and vice versa. Sample size was calculated using Pass Software [31] with the formula for power analysis of cluster-randomized non-inferiority for the ratio of two independent proportions. A non-inferiority margin was chosen a priori, which assumed acceptability of non-treated clothing was 95% and the maximum difference in acceptability between the groups (ITC and NTC) was 10%.

A scenario was selected whereby a minimum sample required 8 clusters per arm and 12 individuals per cluster (192 individuals across the two arms) in order to have >90% power to reject the null hypothesis, assuming an intra-class correlation coefficient (ICC) due to clustering of individuals of 0.002 and alpha of 0.05.

#### Qualitative

Participants in the qualitative study were invited to participate through purposive and convenience sampling. Gatekeepers included basic health staff, key community members (e.g. a village shopkeeper) and the quantitative data collectors, who identified and invited participants during the household surveys. A total of 42 FGDs and 5 IDIs were conducted with rubber tappers enrolled in the trial. FGDs were conducted separately by sex and cluster, with 6–10 participants per group.

### Definitions of non-inferiority and acceptability

‘Non-inferiority’ of ITC can be interpreted as meaning ITC is considered ‘no worse than’ regular, untreated clothing (NTC). To determine this, participants were asked to rate the clothing (yes/no) against a number of acceptability indicators which were designed to measure perceptions towards the clothing’s aesthetic characteristics and practical attributes, and their willingness to recommend or buy it (clothing looks nice; clothing is pleasant to wear for night-time work clothing is durable; clothing is easy to clean; clothing reduces mosquito bites; would recommend clothing; would buy clothing if available in the market; overall, like the clothing). Acceptability was concluded if most of these indicators revealed non-inferiority of ITC to NTC (Table 2).

### Intervention

**Fig. 3:**
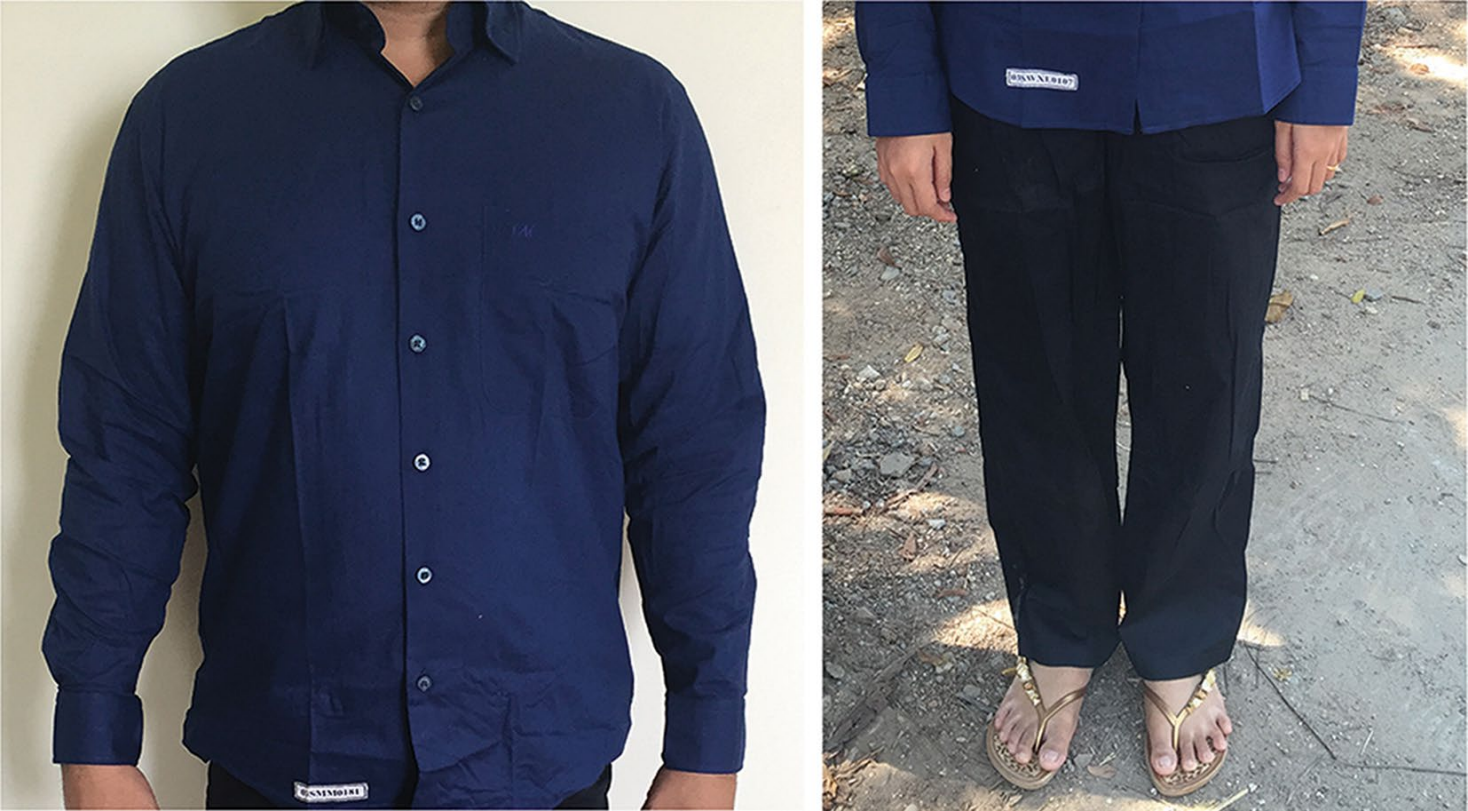
Insecticide-treated clothing distributed as part of the intervention. *Left* long-sleeved navy *blue cotton shirt*. *Right*: *long black cotton trousers*. During the sub-study (September–October 2015), shirts and trousers were both *navy blue* in colour due to supply limitations. ITC and NTC were identical apart from presence of insecticide

Enrolled rubber tappers received ITC and NTC in four standard Myanmar sizes (Fig. 3). ITC was a set of long sleeve cotton shirt and long cotton trousers, Myanmar-made and purchased from a local market and treated with a long-lasting permethrin formulation using a factory proprietary method containing 0.52% w/w ± 10% permethrin and a polymer (Insect Shield; United States Environmental Protection Agency registered and WHO-approved). NTC was identical but untreated and odourless.

### Procedure

**Fig. 4:**
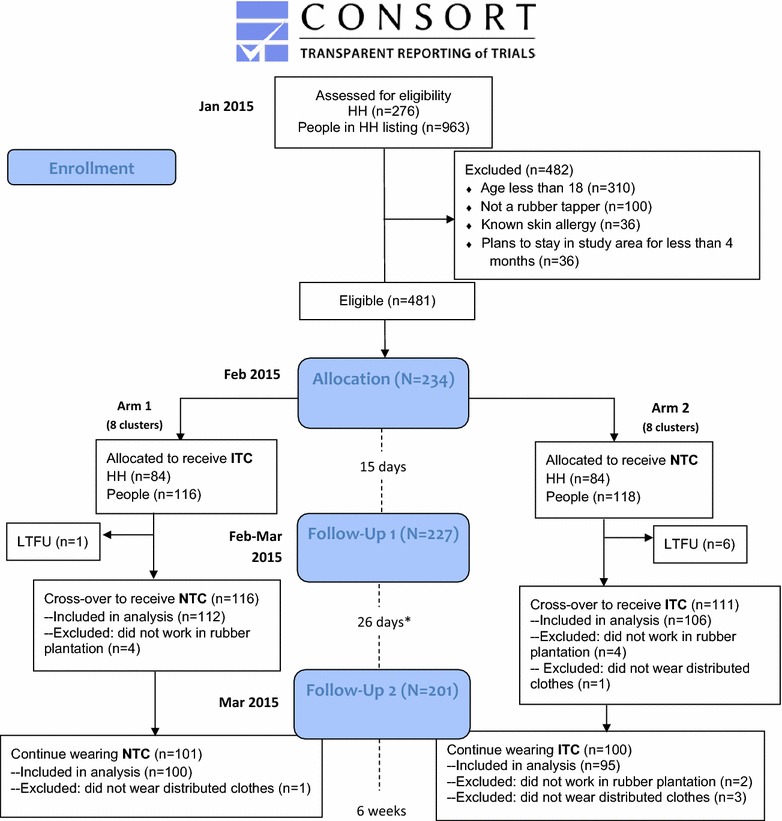
Participant flow according to CONSORT 2010 Flow Diagram. *Timing of second follow-up was later than intended due to postponement of field visit due to unavailability of field assistants (interval should have been 15 days). The order in which ITC and NTC were trialled was randomly assigned by cluster (8 clusters assigned to Arm 1; 8 clusters assigned to Arm 2). Participants within a cluster received the same type of clothing (ITC/NTC) to trial at the same time, and were blinded to the order in which clothing was distributed. Clusters in Arm 1 were assigned to trial ITC followed by NTC. Clusters in Arm 1 trialled NTC followed by ITC

A baseline survey and mini-census were conducted in January 2015 to identify eligible participants and define clusters. Screened participants were enrolled according to the sampling frame and clusters were randomized to the sequence in which the two types of clothing (ITC and NTC) were tested. Clusters in Arm 1 were assigned to trial ITC followed by NTC, and clusters in Arm 2 trialled NTC followed by ITC. Participants were blinded to the order in which ITC and NTC were distributed. At each follow-up visit (follow-ups 1–3), a short acceptability survey was administered to each household. FGDs were conducted with a small sample of participants at baseline and every follow-up visit, and five IDIs were also conducted at the third follow-up visit. Household interviews, FGDs and IDIs were conducted in Burmese by trained researchers using a pre-tested questionnaire or topic guide. Participant flow is illustrated in Fig. 4.

## Methods

### Quantitative methodology

Quantitative data was collected through face-to-face interviews, recorded by hand on paper questionnaires at the time of interview and checked for completeness and inconsistencies before leaving the household, and again at the rest house. Data was double-entered in EpiData version 3.1 (EpiData Association, Odense, Denmark) in Yangon.

The baseline questionnaire collected information on sociodemographic characteristics, current malaria prevention and personal protection methods, current clothing use, and clothing preferences. Information on wealth quintile and socioeconomic status was collected at Follow-up 1. The questionnaire used at Follow-up 1 and 2 collected information on distributed clothing use patterns, care practices, perceptions and acceptability, clothing survivorship and adverse events. Follow-up 3 also investigated the existence of preference for one type of distributed clothing over another.

### Quantitative data management and analysis

Data cleaning and analysis were carried out using Stata version 13 [32]. Simple proportions and means were computed to describe study parameters, with Chi square and t tests used to determine differences between study arms at baseline. Households were classified by wealth quintiles using a principal components analysis of household assets data according to standard Demographic and Health Survey (DHS) methodology [33]. To assess the non-inferiority of ITC over NTC, a cluster level analysis of data collected during follow-up visits 1 and 2 was carried out [34]. For each acceptability indicator, the mean absolute difference between ITC and NTC was estimated using ordinary least squares regression (OLS), adjusting for the order in which the clothing was distributed (ITC followed by NTC, or NTC followed by ITC) and cluster size. The 95% confidence interval (95% CI) of the mean difference was used to determine non-inferiority, using a pre-specified non-inferiority margin of 10%, where a difference of less than or equal to 10% between the two groups was considered to be of public health importance. Non-inferiority is indicated if the 95% CI around the mean difference falls below the pre-defined non-inferiority margin of 10%.

### Qualitative methodology

FGDs and IDIs were recorded using a digital recording device, transcribed verbatim on the same day and field notes incorporated. Audio recordings were destroyed immediately after transcription. Transcripts were later translated from Burmese to English language for content analysis.

The qualitative study complemented the quantitative questionnaires by exploring key topics relating to clothing perceptions, preference, acceptability and use in more depth. These topics were:

1. Knowledge about malaria, prevention of malaria and clothing preferences (Baselines 1 and 2)
2. Preferences and acceptability of distributed clothing (at follow-up surveys 2, 3 and 4)
3. Use and maintenance of distributed clothing (at follow-up surveys 1, 2, 3 and 4)
4. Suggestions for improvement to clothing (at follow-up surveys 3 and 4).

### Qualitative data management and analysis

Topic guides provided an initial thematic framework for analysis, however some themes and sub-themes emerged during the data collection which were verified with other data collectors and incorporated in the topic guides accordingly; two researchers then coded the English transcripts through line-by-line content analysis, identified emergent themes and sub-themes, and verified these with one another, and local members of the research team, to gain multiple perspectives and context and minimize bias. All data was sorted and organized using the thematic framework in Excel 2010^®^ and patterns, similarities and differences identified between the strata before being contextualized by theme. The qualitative data were validated with different respondent groups, analysts and methods i.e. FGDs, IDIs and informal observations in the field. The qualitative and quantitative data were triangulated to complement the findings, improve completeness and facilitate deeper understanding of the data.

### Adverse events

Participants were informed about the (relatively low) risk of experiencing adverse events and were instructed to report to their responsible basic health staff in the instance of experiencing any symptoms. Trained basic health staff from the rural health centre and sub-rural health centre responded to cases and collected this data according to a standard template.

## Results

### Baseline demographics

Overall, 276 households with 963 individuals were assessed for eligibility. Of these, 108 households were ineligible to participate (Fig. 4). There were no significant differences found between participants meeting study eligibility criteria and those that did not participate. One hundred and sixty-eight households (234 individuals comprising 138 heads of households and 96 other family members) in 16 clusters completed the questionnaire at baseline. Clusters ranged in size from 7 to 25 individuals. Clusters (comprising 234 individuals) were randomly assigned to one of the allocation arms and followed up at regular intervals to assess use, frequency of wear, and acceptability of the distributed clothing (see Fig. 4 for participant flow; 7 participants lost between baseline and first follow-up).

There were no significant differences in age, gender, education, and geographic origin of the household respondent at baseline (Table 1). The mean age of household respondents was 33 years. Over 60% of the respondents were male and attended up to the primary school level. Most respondents were from in-state (i.e. Mon State), with out-of-state rubber tappers coming from mostly the Bago and Irrawaddy regions.

**Table 1:** Baseline demographics of households by trial arm

	Arm 1 (ITC then NTC) (n = 84 households)	Arm 2 (NTC then ITC) (n = 84 households)
Respondent (n, %)		
Head	51 (61%)	51 (61%)
Other	33 (39%)	33 (39%)
Mean age (n ± SD)	33 ± 11	33 ± 11
Gender (n, %)		
Male	52 (62%)	56 (67%)
Female	32 (38%)	28 (33%)
Mean household size (n ± SD)	3.9 ± 2.0	3.5 ± 1.5
Education (n, %)		
Primary or lower	55 (65%)	51 (61%)
Middle	16 (19%)	21 (25%)
Secondary or higher	13 (16%)	12 (14%)
Wealth quintile*		
Highest	12 (14%)	22 (26%)
Fourth	12 (14%)	22 (26%)
Middle	17 (20%)	16 (19%)
Second	18 (21%)	16 (19%)
Lowest	25 (30%)	8 (10%)
Distance (miles) to the nearest health centre (n ± SD)	3.0 ± 10.7	1.9 ± 2.2
Geographic origin (n, %)		
In-state (Mon)	49 (58%)	57 (68%)
Out of state (Bago, Irrawaddy, Other)	35 (42%)	27 (32%)
Plans after rubber tapping season (n, %)		
Remain at current plantation	45 (54%)	44 (52%)
Return home	36 (43%)	37 (44%)
Work at other plantation	3 (3%)	3 (4%)
Malaria prevention methods used**		
LLIN*	40 (48%)	53 (63%)
Mosquito coil	38 (45%)	36 (43%)
Wood smoke*	13 (15%)	4 (5%)
Repellent	5 (6%)	4 (5%)

* Chi square test indicate significant difference of p < 0.05 between arms

** Multiple responses indicated, only top answers presented

Wealth quintile and methods of malaria prevention used at baseline varied between arms, but were controlled for in the cross-over analyses. Households in Arm 1 (trialled ITC then NTC) were more likely to be poor (30% in the lowest wealth quintile compared to 10% in Arm 2 (trialled NTC then ITC), p < 0.05). Respondents in Arm 2 were also more likely to use LLIN as a malaria prevention method (p < 0.05), and a higher proportion of respondents in Arm 1 said they used wood smoke as a prevention method compared to those in Arm 2 (p < 0.05).

### Baseline malaria knowledge and personal protection methods

#### Quantitative

The most commonly used methods of mosquito prevention reported by participants in the baseline survey were LLIN (56% across both arms) and mosquito coils (44% across both arms).

#### Qualitative

Baseline malaria awareness and knowledge was generally good: participants could correctly list symptoms and mosquitoes or mosquito bites were commonly cited as the cause of malaria. However, misperceptions about the cause of malaria were also prevalent, such as drinking or bathing in unclean or fresh water, eating certain foods, and poor hygiene and sanitation.

In the FGDs, participants described a wider range of methods for mosquito prevention, including modifications to existing methods. In particular, the participants from one village described using mosquito coils tucked into a headband or waistband during their night-time work. A number of participants also mentioned wearing long clothing (long sleeves; *longyi* or *htamein* [traditional sarong]) to prevent mosquito bites, and a few mentioned cleaning surroundings, streams and water sources, and emptying water vessels.

“We lit the mosquito coils on the head (showing how to do it with their hands). Some [boys] also took [the mosquito coil] with the long stick in their waist” – FGD, female rubber tappers

Mosquito repellents and creams were not commonly used and were not popular due to the odour and fear of toxicity or adverse events.

“I didn’t use [the repellent cream] because it is not suitable for the body as it is the poison.” FGD, male rubber tapper

### Baseline clothing worn and preference (pre-intervention)

#### Quantitative

Despite some demographic differences between study arms at baseline, there were no significant differences in clothing used or clothing preferences at baseline. Over half of the respondents typically wore dark clothing (64% wearing navy blue or black clothes) and 67% wore long clothing to work (43% wear both long shirts and trousers; 26% wear a long shirt, and 2% wear long trousers). Of those that did not wear long clothes for work (n = 56), 50% cited ‘*too hot’* and 20% cited ‘*not having long clothes available’* as reasons for not wearing long clothes for work.

At baseline, clothing preferences of participants were similar to the usual clothing worn. Half of participants (54%, 91 out of 168 households) preferred to wear black clothing, followed by navy blue (13%). The most common style was long shirt plus long trousers (43%) followed by a long shirt plus short trousers (24%). The most commonly cited reasons for these preferred styles were that they were easy to work in (38%), prevented insect bites (37%) and were comfortable (18%).

#### Qualitative

Generally, qualitative findings were in line with the quantitative results. Some of the key findings on clothing preferences pre-intervention are outlined below.

##### Comfort and flexibility

Most participants explained that they liked their work wear to be comfortable, easy to move in and breathable. Soft, elastic textures and cotton were typically preferred (flannel was popular among women).

“We want soft texture clothing so we can sit and stand freely; we don’t need to worry about it being torn. Our job nature is moving, sitting and standing frequently so we want elastic (loose) texture type clothing.” FGD, male rubber tapper

“Nylon is hot. Cotton is cool and comfortable for working and moving. It’s freely moving as we like.” FGD, female rubber tapper

A few participants said they liked to wear thicker material to prevent mosquito bites, but this was reported by some to be less comfortable. Male and female participants preferred loose-fitting clothing, though men specifically mentioned no buttons and a sport suit type design. *“I wear the thick shirts for not to be bitten by the mosquitoes.” FGD, male rubber tapper*

##### Long clothing

The majority of male participants said they work in long sleeved shirts and long pants, with a few alternating between wearing long pants and a *longyi* (male sarong), with boots. Women wore a wider range of work outfits, including long and short sleeved shirts, long and short pants, *longyi/htamein* (female sarong), scarves (for warmth), socks and long rubber boots, for additional protection against scratches, snakes and scorpions in the rubber forest. Some participants expressed a preference for long clothing because they said it could prevent mosquito bites. Traditional *longyi* or *htamein* were more likely to be worn by the older generation, although trousers were generally said to be a more convenient style for rubber tapping.

“I wear htamein. They are young so they wear long pants.” FGD, female rubber tapper

Weather was also a factor; long sleeves and pants were preferred for cooler weather and short sleeves and short pants for warmer weather.

“When the weather is hot, we wear short pants. When the weather is cold, we wear long pants.” FGD, female rubber tapper

##### Dark colours

All participants reported that they preferred to wear dark colours to hide dirt and stains from the rubber latex which accumulates during work.

“We choose the dark colour and chequered design, as these won’t be stained. The light colour is easily stained.”- FGD, female rubber tappers

##### Low quality

Participants explained that they work in “the clothes that are worth the job”, that is, not expensive or high quality, due to the rapid wear and tear, staining and overall short lifetime of the clothing (usually 1–8 months).

“We can’t wear the nice one [clothing]. If we wear like this, the cloth is stained by the rubber liquid. So, the nice fancy one is not suitable for wearing.”- FGD, male rubber tapper

“We only wear the rubber tapping clothing for one month [before throwing it out]. That is why we do not need expensive clothing (laughing).”- FGD, male rubber tappers

##### Multiple sets

Most participants owned at least two sets of work clothing (one as a spare) due to the need for regular washing. Many also kept a spare, clean set to change into after work. They explained that they clothing does not last very long and needs to be replaced regularly.

“After work, we wash the clothing. We need to wash every day because of bad odour. Sweating a lot during working and it makes bad odour.” – FGD, male rubber tapper

“We have two pairs of clothing for rubber tapping. These clothing are easily stained with rubber liquid.” –FGD, female rubber tappers

### Follow-up results

Data from the first and second follow up visits, conducted in February and March 2015, are presented below.

### Adherence

Participants were asked about the number of times they wore the clothing in the preceding period at each follow up visit.

#### Quantitative

Frequency of use was similar across clothing types; people wore the clothing on an average of 11 nights in the previous two weeks. Approximately 80% of participants wore the distributed clothes only. There was a significant difference in the likelihood to wear distributed clothing every night: 80% of participants wearing NTC were likely to wear it every night compared to 70% in ITC (p < 0.05). The most common reasons for not wearing the distributed clothing every night were that participants were washing or drying the clothing (approximately 33%) or did not work in the plantation that night (approximately 40%).

#### Qualitative

A majority of participants reported wearing the clothing regularly for night-time work. In addition to this, some participants said that they wore the clothing at other times of the day, for example when going to town or sleeping. Protection against mosquito bites was a major reason given for wearing the clothing.

“Previously we wore short sleeve shirts and short pants, now we wear the distributed clothing [because] it protects from mosquito biting.” FGD, male rubber tapper

The main reasons given for not wearing the distributed clothing were: it was the wrong size, the clothing was too hot, or it was being washed or dried. A small number of participants experienced damage or tears in the clothing and stopped wearing it until it was repaired.

“In the first week I wore it, but I can’t wear it now as the weather is too hot.” – Female rubber tapper

“My clothing is not dry after washing, so I do not wear on some nights.” – FGD, male rubber tapper.

### Acceptability

#### Quantitative

Participants were asked their opinion on a number of acceptability indicators for each distribution round. Table 2 presents the acceptability of ITC and NTC (aggregated across distribution rounds), the mean difference, and 95% confidence intervals around the mean differences across a number of dimensions of acceptability. In general, participants had very similar opinions about ITC and NTC. A large majority (>89%) of participants who received NTC and ITC across the distribution rounds believed that both types of clothing looked nice, were pleasant to wear for night-time work, and were durable and easy to clean. A high proportion of participants also believed that both ITC and NTC reduced mosquito bites (98 and 94%, respectively), and a majority reported that this was the characteristic they most liked about the clothing (61% of those wearing ITC; 63% of those wearing NTC).

**Table 2:** Non-inferiority of ITC relative to NTC

Indicator	Distribution 1				Distribution 2				Difference (NTC-ITC)		Conclusion of non-inferiority
	ITC Arm 1 (n = 116)		NTC Arm 2 (n = 111)		ITC Arm 2 (n = 100)		NTC Arm 1 (n = 101)		Mean absolute difference*	95% CI	
	N	%	N	%	N	%	N	%			
Clothing looks nice	107	97	96	95	92	93	94	95	1.2	[−4.7 to 7.0]	Yes
Clothing is pleasant to wear for nighttime work	97	93	84	93	88	98	98	83	−4.7	[−12.2 to 2.8]	Yes
Clothing is durable	109	97	104	99	92	100	100	100	−1.0	[−3.8 to 1.9]	Yes
Clothing is easy to clean	111	96	106	100	94	96	100	100	4.3	[−1.3 to 10.0]	Inconclusive
Clothing reduces mosquito bites	109	97	104	99	93	99	99	90	0.0	[−7.4 to 7.6]	Yes
Would recommend clothing to family and friends	112	97	106	100	94	99	100	100	1.0	[−2.1 to 4.2]	Yes
Would buy clothing if available in market	112	88	106	94	94	96	100	89	−1.3	[−9.1 to 6.3]	Yes
Overall, likes the clothing	112	97	106	99	94	99	100	99	0.0	[−3.1 to 3.3]	Yes

Non-inferiority is indicated if the upper 95% CI around the mean difference falls below the pre-defined non-inferiority margin of 10%

* From OLS logistic regression controlling for order and cluster size

As the difference and the upper 95% confidence interval for the majority of acceptability indicators are below the pre-specified non-inferiority margin of 10%, we conclude that the acceptability of ITC was non-inferior to NTC amongst this study population. Non-inferiority could not be concluded for one indicator (“easy to clean”)—however this was not a primary outcome of the assessment.

A high proportion of participants liked the texture of the distributed clothing (98%), and the colour of the pants and shirt (89–98%). Approximately 60% of participants said that the characteristic they most liked about the distributed clothing (either ITC or NTC) was that it reduced/prevented mosquito bites, whereas 30% said that the characteristic they most liked about the clothing was that it was comfortable to work in.

#### Qualitative

Participants across the groups generally reported liking both the NTC and ITC, but shared positive and negative perceptions in roughly equal proportion. In general, respondents could not detect a difference between ITC and NTC in terms of mosquito protection.

##### Positive perceptions about distributed clothing

A widespread observation made by participants was that the clothing (both NTC and ITC) prevented mosquito (and other insect) bites and reduced the presence of mosquitoes flying nearby, allowing them to concentrate on their work and be more productive. Many participants also commented on the health benefits of the clothing. Both of these factors were associated with participants feeling that they could be more productive and work more.

“Mosquito will not come close to me while wearing the distributed clothing. I can save my time and I can work more because of this clothing.”- FGD, female rubber tapper

“The clothing makes less mosquitoes bite. And then it is good for wearing and also good for health.” FGD, male rubber tapper

Some participants compared the clothing to other preventive measures and commented that wearing the distributed clothing eliminated the need to use other preventive measures against mosquitoes, some suggesting that they perceived it to be superior to existing measures.

“When we wear the distributed clothing, no other measures are needed. It is perfect. In the past, we used mosquito repellent stick putting on the head. We also used mosquito repellent cream.” – FGD, female rubber tapper

A majority of participants described the clothing as comfortable to work in, good quality and well made. Freedom of movement and durability during rubber tapping were regarded as particularly important, as were the weight or temperature of the clothing and quickness to dry, given the need for regular washing.

“The texture of the distributed clothing is good. I can sit and stand freely. It is also suitable for low level rubber tapping (bottom of the trunk).” – FGD, male rubber tapper

##### Negative perceptions about the distributed clothing

While participants generally liked the clothing, the most commonly cited problems were that it was the wrong size or too hot to wear (more frequently reported during the drier months of the study).

“The size of the shirt is too small for me to wear. I can’t cut the rubber stem as it is tight at the armpit.” - FGD, female rubber tappers

Feedback suggested that ‘free-size’ or one-size-fits-all clothing with an elasticated waistband and no buttons (hooks suggested instead) would be preferred and could overcome the issue of poor fit.

“Being workers like us, we like pull-over shirts more.” FGD, male rubber tapper

Although during the winter months, participants commented that they found the clothing to be cool and comfortable (“It is good and not feeling hot.”—FGD, female rubber tapper), this trend changed as the study moved into the dry season. Participants then more commonly reported that the clothing was too hot to wear or that they had not worn a full set.

“It is not comfortable with this season; weather is hot. I did not wear the shirt.”- FGD, male rubber tapper

A small number of participants expressed concern about the insecticide and perceived risk of side effects.

“For me… I worry about baby if I am pregnant. It can cause poisoning.” – FGD, female rubber tapper.

### Affordability and willingness to buy the clothing

#### Quantitative

Ninety-three percent of those wearing ITC and 91% of those wearing NTC would buy the clothing if it were available in the market. In general, the amount individuals were willing to spend increased with education level (p = 0.02) (Table 3).

**Table 3:** Amount participants* would be willing to spend on the clothing, by education level

	Total, n (%)	Illiterate/read and write, n (%)	Primary, n (%)	Middle, n (%)	High + , n (%)	p**
Amount willing to spend by category, Myanmar kyats						
1000–3999	70 (18.7)	20 (28.2)	31 (18.5)	8 (9.4)	11 (21.6)	0.02
4000–6999	203 (54.1)	39 (54.9)	93 (55.4)	49 (57.7)	22 (43.1)	
7000–9999	43 (11.5)	7 (9.9)	21 (12.5)	7 (8.2)	8 (15.7)	
10,000+	59 (15.7)	5 (7.0)	23 (13.7)	21 (24.7)	10 (19.6)	
Total	375	71	168	85	51	

* Those who said they would be willing to buy the clothing if it were available in the market

** From Chi squared test for significance

#### Qualitative

Most participants said they would be willing to pay between MMK 3000 ando 5000 (approximately USD 3–5 at the time of the trial) for a set of clothing, but that they would be prepared to pay more if it was of better quality or had a unique and proven purpose, such as preventing mosquito bites. However, many requested the clothing to be provided free-of-charge, given their limited disposable income after purchasing other necessities such as food and work supplies. Participants, plantation owners and managers also said they had been affected by falling global rubber prices (since the onset of the global financial crisis in 2008), which impacted on their job security and the amount they would be willing or able to spend.

“We can spend about 4000-5000 kyats [on the clothing] at the most. We got less money as daily wage [since the rubber market crashed]. There is no left-over money as we [have to] buy the other essential things like food and so on…” – FGD, female rubber tappers

“I will only buy these if it makes relief from mosquito’s bite. (Laughing) If it is useful, I will buy surely.”– FGD, male rubber tapper

### Demand for the clothing and willingness to recommend it

Both the quantitative and qualitative findings revealed that almost all participants would recommend both types of clothing to their family and friends (some had already done so) and liked the clothing overall. Findings suggested there is community demand for clothing that can offer health benefits, such as ITC.

“I told my friends like that the distributed clothing are very good for my health and it is good to wear. They want to buy after my talk, they asked me if they can buy this clothing at market.” –FGD, male rubber tapper.

### Adverse events

No serious adverse events were observed in the study. Reports were made by 5% (n = 21) of participants and these were mild in nature, ranging from sneezing, skin irritation, general itchiness, and headaches. Of these 21 cases, six were reported among participants wearing NTC and can therefore be excluded as not being attributable to the intervention.

“After wearing [the ITC] for 4 hours, I got dizziness. I took off the shirt and put at my waist.” – FGD, male rubber tapper

### Participants’ suggestions for improvement

The qualitative component allowed for further exploration of participants’ perceptions and suggestions for improvement to the clothing and the ITC strategy. Key suggestions included developing promotional and educational messages and delivering these through mass media channels, such as billboards and the radio; ensuring access for rubber tappers by distributing ITC in rural areas and through health centres and shops, and regularly at key intervals throughout the year, such as the start of the rubber season and when mosquito density is highest. Participants also commented on the difficulty of correct sizing, and suggested taking measurements before clothing distribution, designing clothes to be ‘free size’, or treating their own clothing with insecticide. Promoting two types of clothing (thick and thin) was also suggested to improve adherence year-round, in the cool and the dry seasons.

## Discussion

This trial showed that among a migrant rubber tapping population in Mon State, acceptability of ITC was non-inferior to NTC. The high acceptability for both types of clothing in this trial was reflected in the strong adherence to the intervention, participants’ willingness to buy and recommend the clothing, and high ratings given for the acceptability indicators. Importantly, the clothing was also perceived to have a function in personal protection against mosquito bites.

### Mosquito prevention and personal protection

The low use of traditional preventive measures such as LLIN at baseline, coupled with the prevalence of modified methods, such as mosquito coils tucked into a headband or belt buckle during night-time work, illustrates the perceived need for personal protection in the rubber plantation. A large percentage of participants (96%) believed that the distributed clothing prevented mosquito bites, and many cited this reason to explain why they wore the clothing regularly, including at other times of the day. A few participants commented that wearing the distributed clothing eliminated the need to use other or current protection methods, and highlighted the practical advantages, including a positive impact on their productivity and health. These findings suggest that ITC could suitably and acceptably fill a gap in personal protection for this outdoor occupational risk group.

### Willingness to recommend the clothing to family and friends

A high proportion of participants (98% wearing ITC; 100% wearing NTC) were willing to recommend the clothing to family and friends and this remained constant over the course of the trial (Table 2). The qualitative findings also revealed there to be demand in the community for clothing that can provide personal protection or health benefits. The participant who explained how his friends had been eager to buy the clothing for themselves after hearing him advocate about it, is one striking example of the power of word-of-mouth in small communities. Identifying individuals who are influential and can act as role models within the community through a positive deviance approach has been shown to have promise in malaria control and elimination settings in the GMS [35]. Future trials or ITC distribution schemes should seek to harness these individuals and utilize community mobilization approaches to ensure sustainability of the strategy.

### Willingness to buy the clothing and cost considerations

Most of the participants (93% wearing ITC; 91% wearing NTC) said they would be willing to buy the clothing if it was available in the market, and many supported this by citing the health benefits. However, in general, participants expressed reservations about cost, suggesting that free distribution would be preferable given their limited discretionary income. Similar reservations about payment (despite willingness to recommend) were also reported in a study on acceptability of insecticide-treated school uniforms in Thailand [29] and are a potential obstacle to feasibility. Subsidization schemes and costing mechanisms to address the gap between willingness and ability to pay were recommended in a supply and demand analysis conducted as part of this study [36] and have been put forward in other trials of insecticide-treated materials [29, 37]. Further details on cost of ITC and potential financing mechanisms will be discussed in an upcoming paper (Jeffrey Hii, personal communication, 2016).

### Participants’ preferences and suggestions for improvement

Participants suggested that a pullover or ‘free-size’ outfit, with trousers and made of long, loose-fitting material in dark colours would be preferred, to ensure comfort and swift movement at work, hide stains and provide protection against mosquitoes, scratches and other nuisances in the rubber forest. The main reasons for not wearing the clothing related to size, temperature and the frequent need to wash, and for these reasons participants recommended distributing multiple sets of season-appropriate attire. Similar issues with bed nets were voiced by workers in rubber plantation sites in Mon State and Tanintharyi region, who reported sleeping outside of the net on occasion due to the hot weather and work and behavioural factors [38].

The existence of specific clothing preferences identified among the rubber tappers in this study illustrates the need for tailored approaches when introducing a novel tool such as ITC. While the insecticidal activity and effectiveness of ITC is essential to its function as a personal protective measure, it may not be enough to ensure uptake. From a social perspective we must consider what aesthetic and practical characteristics of clothing people want and care about, to ensure they wear it (or don’t object to wearing it), and recognize that these preferences may vary between individuals and populations. In this trial, the scoping assessment performed in 2013 (Muhammad Shafique, personal communication, 2013) provided initial information on working habits and preferred clothing style, colour and material which enabled appropriate clothing to be selected for the intervention according to the target group’s preferences and may be one explanation for why observed levels of acceptability were so high. ITC should be designed to match closely with individuals’ preferences or current clothing style in order to facilitate its adoption. The study revealed that participants considered the treated clothing to be non-inferior to (‘no worse than’) otherwise identical, untreated clothing, with the additional benefit of being able to prevent mosquito bites.

### Feasibility

Worth considering is whether the additional cost and time required for conducting community consultations be sufficiently justified by the increase in uptake of ITC, and whether this is realistic and achievable within the timeline of malaria elimination by 2030 in the GMS. Such tailored approaches can be highly labour-intensive and costly, particularly in a malaria elimination setting (where funding inevitably declines after successes in control). Another potential barrier to feasibility of the ITC strategy among this particular target group is the relatively short lifetime of rubber tapping clothing (due to heavy staining), meaning that multiple sets would be required per person throughout a season. This factor makes it increasingly challenging to find a cost-effective strategy, or to justify tailoring and treating clothing with long-lasting methods, when the clothing itself may only last up to one month in the field. Further research into the optimal treatment method for use in rural settings and at the community and individual level is recommended, and has also been suggested elsewhere [14, 28]. Nonetheless, if employers can be encouraged to provide and promote insecticide-treated uniforms for their workers, this strategy may be successful among specific occupational groups, assuming supportive financing mechanisms are available. Plantation management should be engaged to determine whether there is any potential for their support in subsidizing or purchasing ITC for their workers.

### Seasonal and entomological factors

An ancillary study (not shown) was conducted from September–October 2015 to investigate the influence of seasonality on the findings and the hypothesis that greater acceptability of ITC over NTC might be apparent during the post-monsoon season, when mosquito density was expected to be higher. Based on entomological laboratory observations, no evidence was found supporting this (paper in preparation).

However, the authors suggest that future studies should be conducted during the wet or post-monsoon seasons and in areas with high reported mosquito density to explore this potential effect in detail.

### Limitations

#### Acceptability

Acceptability: It is not possible to determine whether the highly positive acceptability indicators are representative of real-life settings. Monitoring markers of acceptability over a longer time course (outside of trial conditions) is needed, as these have been shown to wane with time [39]. Acceptability may also be inflated during a period of initial exposure to a new intervention, or through response or social desirability bias during a trial, which could have implications for the feasibility (and ethics?) of taking an intervention to scale in the long-term. Future research should also seek to determine whether participants would opt to wear or purchase ITC in instances where it is not given to them, as this would help to elucidate its true perceived value, however this was beyond the scope of the study and would likely require accompanying behavioural change interventions to be realistically evaluated in this setting.

#### Time intervals

The time between the first and second follow-up visit was two weeks longer than scheduled due to public holidays and unavailability of local gatekeepers, which may have introduced recall bias in responses.

#### Study population

Due to falling global rubber prices, fewer rubber plantations were open and the migrant rubber tapper population was smaller than anticipated. In order to reach the sample size, which had been based on an earlier scoping assessment, PPS sampling was used, with random selection of all females and random selection of males in 3 out of 16 clusters (in the other clusters, all eligible males were enrolled). Also, given that the study was conducted with migrant rubber tappers, caution should be taken before generalizing the findings to other groups.

#### Non-inferiority analysis

Due to a different design, the third follow-up was excluded from the non-inferiority analysis as it was not possible to compare results between rounds. The non-inferiority analysis is therefore only reflective of the first and second follow-ups. The small sample size led to wide confidence intervals in the non-inferiority analysis and also meant that conclusions could not be drawn on superiority of one type of clothing to the other.

### Recommendations

In the first instance, key recommendations are as follows:

1. Conduct further research into bio-efficacy and the optimal treatment method for ITC, particularly for use by rural populations, taking into account lifetime of the clothing, and practical factors such as washing and drying methods.
2. Explore ways to overcome existing barriers relating to cost, through costing mechanisms, such as subsidization strategies, or modifications to the ITC product.

In the future, the following recommendations may be applicable if ITC plans are rolled out:

3. Conduct formative assessments prior to targeted distribution, to tailor ITC to local preferences and encourage uptake.
4. For this target population, provide multiple sets of clothing and seasonal attire, to facilitate adherence and limit gaps in coverage.
5. Utilize local networks for advocacy and distribution, and distribute ITC according to seasonal and occupational patterns.
6. Explore potential strategies for generating demand for ITC, for instance through mass media channels that are accessible and relatable for target populations, or positive deviance and community-based approaches.
7. Develop social and behaviour change communication (SBCC) strategy to promote messages and behaviours related to ITC and deliver these through culturally appropriate channels, such as interpersonal communication through community health workers (CHW), midwives and rubber plantation owners and managers, local media such as TV parlours and loud speakers in the Myanmar context.
8. Use advocacy strategies to engage policy makers, rubber plantation owners and managers, and encourage them to provide and promote ITC for their workers.
9. Establish monitoring and surveillance systems within ITC distribution programmes to target mobile individuals who may arrive at irregular time points and be missed by traditional schemes or distribution schedules.

## Conclusion

Providing communities with personal protective tools that take into account their practices and preferences and are tailored to purpose (e.g. protection specifically during rubber tapping) are needed to target the gaps which traditional core measures cannot reach. This is particularly important in pre-elimination settings such as Myanmar where a targeted approach is required. The high level of acceptability in this study suggests that ITC could be a suitable strategy for personal protection among rubber tappers in outdoor transmission settings (in Myanmar), however more research is needed into cost-effectiveness and potential financing mechanisms, access as well as efficacy and effectiveness of ITC in field settings (see upcoming paper [Jeffrey Hii, personal communication, 2016]), before ITC can be considered for wider roll-out.

## Abbreviations

CDC: United States Centers for Disease Control and Prevention
CHW: community health worker
DHS: demographic and health survey
FGD: focus group discussion
GMS: P Greater Mekong Sub-region
IDI: in-depth interview
IRS: indoor residual spraying
ITC: insecticide-treated clothing
LLIN: long-lasting insecticidal net
NMCP: National Malaria Control Programme
MMK: Myanmar kyat
MMP: mobile and migrant populations
MOH: Ministry of Health
NTC: non-treated clothing
PPS: probability proportional to size
RHC: rural health centre
SBCC: social and behaviour change communication
WHO: World Health Organization
